# Development and psychometric testing of the clinical networks engagement tool

**DOI:** 10.1371/journal.pone.0174056

**Published:** 2017-03-28

**Authors:** Jill M. Norris, Kent G. Hecker, Leora Rabatach, Tom W. Noseworthy, Deborah E. White

**Affiliations:** 1 Faculty of Nursing, University of Calgary, Calgary, Alberta, Canada; 2 Department of Community Health Sciences, Cumming School of Medicine, University of Calgary, Calgary, Alberta, Canada; 3 Department of Veterinary Clinical and Diagnostic Sciences, Faculty of Veterinary Medicine, University of Calgary, Calgary, Alberta, Canada; TNO, NETHERLANDS

## Abstract

**Background:**

Clinical networks are being used widely to facilitate large system transformation in healthcare, by engagement of stakeholders throughout the health system. However, there are no available instruments that measure engagement in these networks.

**Methods:**

The study purpose was to develop and assess the measurement properties of a multiprofessional tool to measure engagement in clinical network initiatives. Based on components of the International Association of Public Participation Spectrum and expert panel review, we developed 40 items for testing. The draft instrument was distributed to 1,668 network stakeholders across different governance levels (leaders, members, support, frontline stakeholders) in 9 strategic clinical networks in Alberta (January to July 2014). With data from 424 completed surveys (25.4% response rate), descriptive statistics, exploratory and confirmatory factor analysis, Pearson correlations, linear regression, multivariate analysis, and Cronbach alpha were conducted to assess reliability and validity of the scores.

**Results:**

Sixteen items were retained in the instrument. Exploratory factor analysis indicated a four-factor solution and accounted for 85.7% of the total variance in engagement with clinical network initiatives: *global engagement*, *inform* (provided with information), *involve* (worked together to address concerns), and *empower* (given final decision-making authority). All subscales demonstrated acceptable reliability (Cronbach alpha 0.87 to 0.99). Both the confirmatory factor analysis and regression analysis confirmed that *inform*, *involve*, and *empower* were all significant predictors of *global engagement*, with *involve* as the strongest predictor. Leaders had higher mean scores than frontline stakeholders, while members and support staff did not differ in mean scores.

**Conclusions:**

This study provided foundational evidence for the use of this tool for assessing engagement in clinical networks. Further work is necessary to evaluate engagement in broader network functions and activities; to assess barriers and facilitators of engagement; and, to elucidate how the maturity of networks and other factors influence engagement.

## Introduction

Large-scale transformation in healthcare requires engaging stakeholders across the health system.[1–4]. Engagement has been described as the active involvement of stakeholders in maintaining and enhancing the performance of their organisation.[2,3] Evidence suggests that when healthcare professionals are engaged in their health system, organizations benefit from reductions in mortality, adverse drug events, errors, and infection rates,[5–8] as well as enhanced quality of care and patient experience.[7,9,10] Findings from a large-scale study in the NHS indicated that trusts with higher staff engagement exhibit better financial performance.[7] Alongside this growing evidence for the link between engagement and performance, there have been a number of advances in physician[3,6,11,12] and patient engagement in healthcare,[13–15] Efforts to engage the spectrum professionals and stakeholders who design and carry out quality improvement initiatives, however—a process outlined within numerous healthcare improvement models.[16–22]—have often been met with limited success.[23–25]

Arguably, one of the major gaps towards improving suboptimal engagement in healthcare improvement is the lack of a tool to measure the process. Appraisals about *who* is sufficiently engaged and *levels* that create meaningful improvements have depended upon subjective judgements to date. We are aware of only one recent cross-sectional study that included a measure of work engagement in a large improvement program,[26] and two studies that assessed physician engagement with minority patients[27] and with service quality.[3] Within the organizational literature, engagement has been measured as a multidimensional concept comprised of psychological states (e.g., vigour), enduring traits (e.g., personality) and behaviours (e.g., involvement).[28–35] In contrast, the healthcare improvement literature predominantly describes engagement as a *process* conducted by the organization,[28] in which stakeholders are encouraged to participate in a range of improvement activities and phases, including priority-setting and sustaining projects long term.[16,19–21]

One model that is potentially applicable to engagement in healthcare improvement is the International Association of Public Participation (IAP2) Spectrum of Public Participation.[36] Multiple healthcare organizations have applied the spectrum with multiprofessional clinician groups in Australia[37] and Canada, as well as to patient groups.[38] Building upon Arnstein’s[39] ladder of participation, later modified by Conner[40] and Pretty[41], the IAP2 spectrum of engagement[36] posits that organizational outcomes improve when stakeholders are included in increasing levels of engagement. This includes setting priorities and decision-making through participatory, transformative and democratic processes.[42] Five levels of engagement processes are outlined within the IAP2 spectrum: *inform*, *consult*, *involve*, *collaborate*, and *empower*. At one end of the spectrum, stakeholders are informed about an initiative; on the other end, they are empowered with final decision-making authority. While promising, the IAP2 has not thus far undergone empirical evaluation. The purpose of this study was to (1) develop a multiprofessional tool based on the IAP2 spectrum to measure engagement processes in clinical network initiatives, and (2) assess the measurement properties of this tool, including an assessment of reliability and validity of the scores.

## Methods

### Context

Over the past decade, health systems globally have implemented clinical networks, and one variant, strategic clinical networks (SCNs), as a means of improving quality of care through a systems-level approach to change.[43–46] SCNs were established in 2012 by Alberta Health Services (AHS), the provincial health system in Alberta, Canada. Health systems in Europe (especially in the UK) along with those in Australia and Canada were early adopters of these networks; more recently, we have seen these networks established in New Zealand,[47] Malawi,[48] and Kuwait.[49] Clinical networks are thought to benefit from communication channels and relationships across multidisciplinary and hierarchical levels in the organization, and intend to achieve meaningful active involvement of various stakeholders.[45,46,50] While clinical networks vary greatly in their focus—whether it be a clinical area, condition, population, operational area, or an intervention—they are all structured to enable better coordination between essential healthcare stakeholders.

We studied engagement in the newly formed SCNs because part of their mandate was to engage partners across the health system. This could provide valuable baseline information to decision makers during the formative and developing stages of these structures. Moreover, AHS had adapted the IAP2 spectrum as their model of clinician engagement. We previously established relationships with the senior decision-makers in AHS, which provided an excellent opportunity to co-design this study and propose a co-developed program of research to evaluate SCNs. This resulted in the adoption of an integrated knowledge approach in which researchers and knowledge-users worked together to craft research questions, refined the methodology, and remained involved in interpreting and using the findings.

### Item construction

The purpose of the tool was to measure perceptions of engagement over various phases of the implementation of projects undertaken by the SCNs. The descriptions and labels within the IAP2 Spectrum[36] first informed the construction of 25 original items for each of the five levels of engagement. Next, we then tailored the item wording to ensure relevance and refer to the activities of the networks, and further worked with our knowledge users to generate an additional 15 items that aimed to capture engagement more broadly than the Spectrum. The 40 items then underwent independent review by a panel of six purposively sampled content experts who had direct experience working with the SCNs, including health services and clinical network researchers, employees of AHS who were working with the SCNs on their engagement strategies, and SCN decision-makers. They were asked to give an assessment of item contents, item style and comprehensiveness of the instrument.[51] In addition, a convenience sample of six clinicians (nurses, physicians) independently reviewed the items before testing the instrument with a larger sample.

Based on the feedback, the research team revised and compiled a final set of 40 items: 4 items that intended to measure the overall (i.e., global) engagement construct (dependent variable) and 36 items that characterized five potential engagement level constructs (inform, consult, involve, collaborate, empower; independent variables). As a response to requests during the item construction stage, *6 = not applicable* option was added for engagement level items, as were items related to evaluating projects. Within each construct grouping, items assessed engagement across four phases of implementation: setting priorities, planning, implementing, and evaluating projects. Overall engagement items were anchored with *1 = not at all engaged* to *5 = extremely engaged*. Engagement-level items were anchored with *1 = strongly disagree* and *5 = strongly agree*. Table A in S1 File lists the original items.

### Instrument testing

#### Participants and procedures

Between January and July 2014, participants were recruited from the nine SCNs purposively selected for participation in this study (Addiction & Mental Health; Bone & Joint Health; Cancer; Critical Care; Cardiovascular Health & Stroke; Diabetes Obesity & Nutrition; Emergency; Senior’s Health; Surgery). Voluntary participation was sought across four levels of the SCN governance structure: (1) leaders; (2) core and working group members, among other stakeholders outside of formalized SCN membership; including, (3) support personnel; and, (4) frontline healthcare stakeholders. Members were identified from SCN membership lists, which included contact information, their position, role on the SCN (e.g., clinician, co-executive leader, zone lead, executive director, working group member), and other organizational contact information. Recruitment occurred first through presentations by the research lead (DW) during SCN meetings. Next, a personalized email describing the study was sent to SCN members. To facilitate response rate, we attended to web survey principles that are effective in achieving higher response rates (e.g., online format, option to complete paper-based survey, simple and usable design, follow-up reminder emails and phone calls at 2- and 4-week intervals).[52]. Eligibility for study participation included the following: a) SCN member or SCN-identified stakeholder outside of the SCN membership; b) English-speaking; and, c) access to a computer.

#### Sample size

We estimated that each SCN would have maximally 100 formal members in the core and working groups from whom to sample, for an estimated pool of 900 SCN members. Using a conservative response rate of 25% for online surveys, a sample of 225 participants would surpass a recommended sample size of 200 participants for both exploratory factor analysis [53,54] and confirmatory factor analysis.[55,56]

### Statistical analysis

Data were first compiled and anonymized, then cleaned prior to analysis. Descriptive statistics were calculated for each item and subscales in SPSS v22 (IBM, Chicago, IL, USA). No values were imputed for missing data.

#### Validation evidence

To examine evidence of construct validity—whether the tool measures the construct(s) intended to assess[57]—we used exploratory factor analysis (EFA), confirmatory factor analysis (CFA), linear regression and one-way multivariate analysis of variance (MANOVA). To test dimensionality of the instrument, an EFA was conducted in SPSS v22 (IBM, Chicago, IL, USA). Unweighted least squares extraction with oblimin rotation and Kaiser normalization was chosen to maximize the variance extracted, given that we assumed that factors would be correlated. To identify the number of factors, eigenvalues > 1 and the scree plot of eigenvalues plotted against factors were examined. Table B in S1 File details the results of the preliminary EFA with all items, which loaded onto six factors, or subscales.

Items that had more than 10% of missing data, cross-loaded (i.e., loads at .32 or greater on two or more factors[58]), or did not load to a factor were explored to determine the conceptual importance of the item, or if inadequately written prior to making a decision whether or not to drop items from further analysis. No items were removed as a result of these inspections. Next, subscale internal consistency and alpha-if-item-deleted were examined, as well as correlations between subscale means for potential multicollinearity (i.e., high correlations between independent variables; *r* > .75). An analysis of internal consistency (Cronbach’s alpha) was completed for the total scale and each of the resulting subscale scores. A Cronbach’s alpha of .70 or greater was considered acceptable. Items that increased the subscale alpha were removed one-by-one from scales that were highly correlated, keeping a minimum of three items that loaded on a factor. Before removal, each item was reviewed (JN, KH, DW) for its potential practical and theoretical implications. In total, 24 items were removed and a final set of 16 items were retained for the final analysis.

To confirm the EFA, a CFA was conducted using EQS 6.1 (Multivariate Software, Encino, CA, USA)[59], in which data were fit to a covariance matrix, using the robust estimation parameter. Fit indices used were the root mean squared error of approximation (RMSEA), and Bentler’s comparative fit index (CFI). Good model fit was indicated by CFI > .95 and RMSEA < .10. A criterion of .40 was set as an acceptable standardized factor loading.

In addition, backward linear regression was employed to determine which factor or combination of factors were predictive of engagement. The dependent variable was *global engagement* and the independent variables were the other resulting subscales from the EFA. One-way multivariate analysis of variance (MANOVA) was used to examine between-group differences of the resulting subscales. We wanted to determine if those who were higher in the governance structure of the SCNs (leaders, formal SCN members) would differ in their levels of engagement compared to those who were more removed from the work of the SCNs (support staff, frontline healthcare stakeholders). We used Games-Howell post-hoc tests as group sizes differed and equal variances were not assumed.[60] Effect sizes were classified by Cohen’s criterion.[61]

### Ethical considerations

Ethical approval was granted by the University of Calgary Conjoint Health Research Ethics Board. Submitting the online survey implied informed consent by respondents. Participants created their own unique identifier for anonymity, and participants’ characteristics have been aggregated to ensure that individuals are not identifiable.

## Results

### Descriptive characteristics of sample

From a total of 1,668 eligible participants within the networks, 424 individuals agreed to participate (25.4% response rate). Participant characteristics are detailed in Table 1. Most respondents were SCN members (55.6%), female (66.7%), older than 50 years (53.3%), and had 25 or more years of professional experience (50.7%). Nearly three quarters of the sample were professional healthcare providers (72.3%), and respondents worked in a wide variety of areas and positions.

**Table 1:** Participant characteristics.

Characteristic		*N*	*%*
**Gender**	Female	283	66.7
	Male	133	31.4
**Age**	18–29 years	5	1.2
	30–39 years	64	15.1
	40–49 years	122	28.8
	50–59 years	175	41.3
	60+ years	51	12.0
**Professional experience**	<5 years	11	2.6
	5–9 years	29	6.8
	10–14 years	37	8.7
	15–19 years	60	14.2
	20–24 years	60	14.2
	25+ years	215	50.7
	N/A	7	1.7
**Professional designation**	Registered nurse, psychiatric nurse, nurse practitioner	141	33.2
	Physician	90	21.2
	Allied health professions staff	78	17.9
	Executive, manager	14	2.9
	Research	13	2.8
	Other	32	6.9
	N/A	37	8.7
**Healthcare provider primary work area (all applicable)**	Acute care	106	25.0
	Surgery	45	10.6
	Emergency	34	8.0
	Outpatient clinic	34	8.0
	Internal medicine	28	6.6
	Community health	25	5.9
	Long-term care	20	4.7
	Psychiatry	20	4.7
	Family medicine	17	4.0
	Primary care	12	2.8
	Neurology	9	2.1
	Social services	3	0.7
	Other	69	16.3
	N/A	96	22.6
**SCN**	Cardiovascular and stroke	77	18.2
	Bone and joint	62	14.6
	Seniors health	53	12.5
	Addictions and mental health	46	10.8
	Surgery	46	10.8
	Diabetes, obesity, and nutrition	44	10.4
	Critical care	39	9.2
	Emergency	30	7.1
	Cancer	27	6.4
**Position (all applicable)**	Medical staff	63	14.9
	Director	51	12
	Patient care manager	35	8.3
	Medical director	33	7.8
	Researcher	33	7.8
	Allied health professions staff	24	5.7
	Executive director	24	5.7
	Educator	23	5.4
	Quality improvement, risk management, patient safety	18	4.2
	Senior executive	18	4.2
	Manager	17	4
	Administration/secretarial/clerical staff	16	3.8
	Nursing staff	14	3.3
	Other	89	20.9
	N/A	47	11.1
**SCN governance level**	Leader	72	17.0
	Member	236	55.6
	Support staff	50	11.8
	Frontline stakeholder	65	15.3

### Data handling–addressing *not applicable* responses

*Not applicable* responses displayed a pattern across items, whereby the proportion of responses increased with items that intended to measure higher levels of engagement (i.e., empower; see Table A in S1 File) and varied significantly by governance level (see Table C in S1 File). Post hoc z tests indicated that the proportion of *not applicable* responses were significantly higher in support staff and stakeholders in comparison to leaders and members (p < .025). We viewed these as important patterns, and subsequently assessed the *not applicable* data through various methods: (a) removal, (b) recoded to bottom of scale, (c) recoded to middle of scale, (d) imputed with the expectation-maximization (EM) algorithm, and (e) none (treated as ordinal-level scale). We compared results of these various methods and decided to conduct an EFA appropriate for non-parametric data with the full sample (*n* = 424), without modifying or removing the *not applicable* response option. Successive analysis used data from which *not applicable* responses were removed (*n* = 310, due to missing data) and data were then treated as parametric, including the calculation of means (*SD*), the CFA to confirm the underlying factor structure from the EFA, correlation, and multiple regression.

### Item distribution

Item descriptive statistics are in Table 2 (for the original 40 items, see S2 File). Means for *inform* items (*M* = 3.35–3.91) were greater than *involve* items (*M* = 3.24–3.32) and *empower* items (*M* = 2.19–2.27). The full range of responses were used for each item and less than 5% had missing data.

**Table 2:** Item descriptive statistics.

Items		M*	SD*	Range	Floor %	Ceiling %	Missing %	N/A %
Q1.	How engaged have you been in the following activities: Setting SCN priorities	2.62	1.32	1–5	27.8	9.4	0.9	-
Q2.	How engaged have you been in the following activities: Planning SCN projects	2.70	1.33	1–5	23.6	11.6	0.2	-
Q3.	How engaged have you been in the following activities: Implementing SCN projects	2.77	1.42	1–5	26.7	15.3	1.2	-
Q4.	How engaged have you been in the following activities: Evaluating SCN projects	2.46	1.37	1–5	34.0	10.4	1.4	-
Q5.	I have been provided with information about SCN priorities	3.91	1.18	1–5	5.2	37.5	0.9	3.3
Q6.	I have been provided with information about how SCN projects are planned	3.50	1.27	1–5	7.8	25.5	0.5	3.5
Q7.	I have been provided with information about how SCN projects are implemented	3.51	1.26	1–5	8.5	24.8	0.9	3.8
Q8.	I have been provided with information about how SCN projects are evaluated	3.35	1.25	1–5	8.0	21.2	1.4	3.1
Q9.	The SCN has worked with me to ensure my concerns and issues have been consistently understood and considered for setting SCN priorities	3.30	1.21	1–5	7.8	14.2	2.1	13.4
Q10.	The SCN has worked with me to ensure my concerns and issues have been consistently understood and considered for planning SCN projects	3.32	1.20	1–5	7.1	14.9	2.6	12.0
Q11.	The SCN has worked with me to ensure my concerns and issues have been consistently understood and considered for implementing SCN projects	3.31	1.18	1–5	6.4	15.1	2.6	12.3
Q12.	The SCN has worked with me to ensure my concerns and issues have been consistently understood and considered for evaluating SCN projects	3.24	1.18	1–5	6.6	13.9	3.3	13.7
Q13.	I have been given final decision-making authority about SCN priorities	2.19	1.10	1–5	23.3	2.8	3.5	22.4
Q14.	I have been given final decision-making authority about how SCN projects are planned	2.23	1.11	1–5	22.6	2.4	3.5	22.2
Q15.	I have been given final decision-making authority about how SCN projects are implemented	2.27	1.12	1–5	21.7	2.6	3.8	21.5
Q16.	I have been given final decision-making authority about how SCN projects are evaluated	2.25	1.08	1–5	22.2	1.9	4.0	21.2

* n = 310

### Exploratory factor analysis

The EFA resulted in a 4-factor solution that accounted for 85.7% of the total variance. Table 3 details the rotated factor loadings, eigenvalues, and percent of variance explained for each factor. The final 16 items clustered on four factors consistent with four of six proposed constructs: *involve* (4 items), *empower* (4 items), *global engagement* (4 items), and *inform* (4 items).

**Table 3:** Exploratory factor analysis of final engagement items.

Items and subscales		Rotated factor loadings			
		1	2	3	4
	**1. Involve**				
Q9.	Involved in setting SCN priorities	**0.84**	-0.01	-0.01	-0.07
Q10.	Involved in how SCN projects are planned	**0.97**	0.02	-0.04	-0.03
Q11.	Involved in how SCN projects are implemented	**0.95**	-0.02	0.03	0.05
Q12.	Involved in how SCN projects are evaluated	**0.94**	-0.04	0.02	0.05
	**2. Empower**			** **	
Q13.	Final decision-making for SCN priorities	0.02	**-0.94**	-0.01	-0.02
Q14.	Final decision-making for how SCN projects are planned	0.03	**-0.97**	0.01	-0.01
Q15.	Final decision-making for how SCN projects are implemented	-0.01	**-0.98**	0.01	-0.03
Q16.	Final decision-making for how SCN projects are evaluated	0.02	**-0.95**	0.01	-0.02
	**3. Global**				** **
Q1.	Engaged in setting SCN priorities	0.11	0.12	**0.48**	-0.25
Q2.	Engaged in planning SCN projects	0.09	0.08	**0.73**	-0.10
Q3.	Engaged in implementing SCN projects	-0.03	-0.07	**0.84**	0.06
Q4.	Engaged in evaluating SCN projects	-0.03	-0.04	**0.91**	0.04
	**4. Inform**				
Q5.	Informed about SCN priorities	0.05	-0.02	-0.07	**-0.82**
Q6.	Informed about how SCN projects are planned	0.01	0.02	-0.05	**-0.96**
Q7.	Informed about how SCN projects are implemented	-0.02	-0.07	0.07	**-0.85**
Q8.	Informed about how SCN projects are evaluated	-0.02	-0.06	0.12	**-0.79**
Eigenvalue		7.94	3.11	1.45	1.20
% of variance		49.65	19.46	9.07	7.50

Note: Rotation converged in 7 iterations; bold font indicates item factor loadings.

### Confirmatory factor analysis

**Fig 1:**
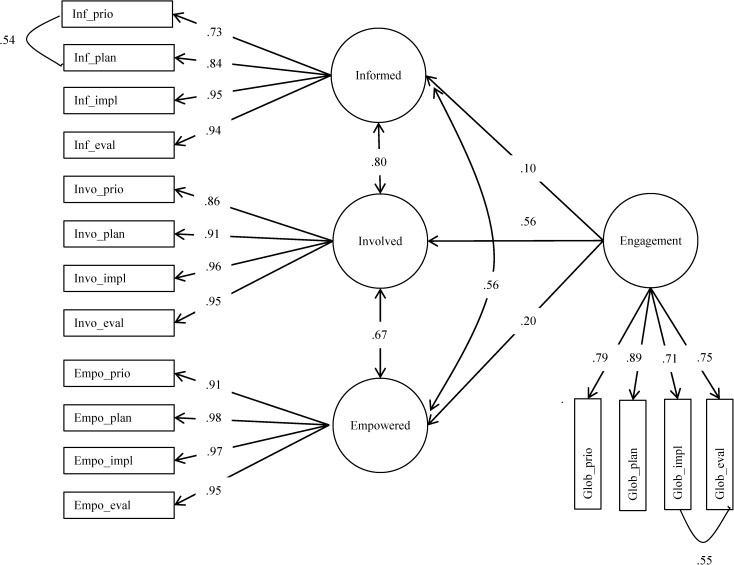
Confirmatory factor analysis results.

Using a CFA, we tested whether the EFA four-factor solution could be replicated after removing the *not applicable* responses. Fig 1 illustrates the results of the CFA, which confirmed the 4-factor structure with good model fit (CFI = .96; RMSEA = .09). Standardized factor loadings were all statistically significant (*p <* .001) and ranged from .71 to .98. *Involve* was the best predictor of global engagement, with a factor loading of .56; *inform* and *empower* were less predictive, with factor loadings of .10 and .20, respectively.

### Scale internal consistency

Analysis of scale internal consistency using Cronbach’s alpha revealed acceptable reliability for all four subscales, which ranged from .87 to .99 (see Table 4). The overall tool achieved a Cronbach’s alpha of .93. Correlations between subscale means were statistically significant (ps < .001) and positive.

**Table 4:** Cronbach’s alpha, means, standard deviations, and correlations between subscale scores.

Subscale	Cronbach’s α	*M*	*SD*	Global	Inform	Involve	Empower
**Global**	0.87	2.64	1.15	-			
**Inform**	0.93	3.57	1.12	.625**	-		
**Involve**	0.96	3.31	1.12	.699**	.791**	-	
**Empower**	0.99	2.24	1.06	.597**	.538**	.635**	-

** *p <* .001.

### Linear regression

Results from the CFA were further confirmed by the regression analysis. Scores from *inform*, *involve*, and *empower* were regressed on *global engagement*. Overall, the level of engagement variables explained 54% of the variance (*R* = .73) in *global engagement*, *F* (3,306) = 109.44, *p <* .001. *Inform* (*B* = .17, *SE B* = .06, β = .17; 95% CI [.04, .29]; *p* = .008), *involve (B* = .41, *SE B* = .07, β = .41; 95% CI [.27, .54]; *p <* .001), and *empower* were significant predictors (*B* = .26, *SE B* = .05, β = .425; 95% CI [.16, .37]; *p <* .001).

### Group comparisons

Governance level had a statistically significant medium effect on engagement scores (*F* (12,802) = 8.61, *p <* .001; Wilk’s Λ = 0.726, *η*^*2*^ = 0.10; see Tables 5 and 6). Significant group differences were found in post-hoc comparisons for each engagement subscale, with mostly medium to large effect sizes. Across all scales, leaders exhibited significantly higher scores than stakeholders (*d =* 1.1 to 1.6, *p <* .001), while members and support staff scores did not significantly differ. Leaders had higher scores than both members and support staff for global *engagement* (*d =* 1.33, 0.90; *p <* .001), *inform* (*d =* 0.64, 0.64, *p <* .01), and *involve* (*d =* 0.73, 0.55; *p <* .05), respectively; leaders also had higher scores than members for *empower* (*d =* 0.63; *p <* .001). In the opposite direction, stakeholders had lower scores than both members and support staff for *global engagement* (*d =* 0.84, 1.02; *p <* .001), and *inform* (*d =* 0.81, 0.70, *p <* .001), *involve* (*d =* 0.71, 0.98; *p <* .001), and *empower* (*d =* 0.41, 1.01; *p <* .05), respectively.

**Table 5:** Means and standard deviations of engagement scores by governance level.

Scale	Leaders (*n* = 72)		Members (*n* = 236)		Support (*n* = 50)		Stakeholders (*n* = 65)	
	*M*	*SD*	*M*	*SD*	*M*	*SD*	*M*	*SD*
**Global**	3.78	1.08	2.51	0.95	2.75	1.06	1.76	0.92
**Inform**	4.24	0.97	3.59	1.02	3.59	1.10	2.69	1.16
**Involve**	4.03	0.96	3.24	1.06	3.49	1.02	2.44	1.05
**Empower**	2.83	1.17	2.13	1.02	2.47	0.94	1.74	0.77

**Table 6:** Multivariate and univariate analysis of variance for engagement scores.

Variable	MANOVA F (12,802)		ANOVA F (3,306)							
			Global		Inform		Involve		Empower	
	*F*	*η*^*2*^	*F*	*η*^*2*^	*F*	*η*^*2*^	*F*	*η*^*2*^	*F*	*η*^*2*^
Governance level	8.61*	0.10	31.79*	0.24	13.37*	0.12	18.72*	.16	9.73*	0.09

* *p <* 0.001.

## Discussion

The objective of this study was to create a brief, multiprofessional tool to measure engagement in SCN initiatives, and establish evidence for reliability and construct validity of the tool. We created items based on the IAP2 spectrum of engagement[36] and included the input of experts and clinicians. From 16 items, four distinct subscales were established through the EFA and CFA: (1) *global engagement*, (2) *inform*, (3) *involve*, and (4) *empower*; all of the subscales demonstrated acceptable reliability. *Inform*, *involve*, and *empower* were all significant predictors of *global engagement*, but both the CFA and regression analysis demonstrated that *involve* was the strongest predictor. Leaders exhibited significantly higher scores across all scales than stakeholders, while members and support staff did not differ in their scores. In sum, we have established preliminary psychometric evidence of this engagement tool for use with SCNs.

The EFA supported three of the five levels within the IAP2 spectrum.[35] Similarly, other models of community engagement have three levels, spanning from concepts that represent one-way information sharing, two-way communication, to shared decision making.[62–64] Although we used direct language from the IAP2 spectrum in developing the items, several members from the expert and clinician panel expressed that there was little differentiation between *involve* and *collaborate* items. This view has been further supported by a critic of the IAP2 who contested that the three middle levels of the spectrum (*consult*, *involve*, *collaborate)* are all “an invitation to selected stakeholders to participate in joint decision making, including the design of the process itself.”[p. 1; 65] As factors loaded by IAP2 levels, our analysis did not support distinctions between four implementation phases: setting priorities, planning, implementing, and evaluating. In the overall sample and governance level groups, there were minimal differences between phase-specific item totals, indicating that participants viewed engagement as interactions with the SCNs instead of involvement in discrete phases. A number of healthcare improvement models would support this, by suggesting that stakeholders should be involved right from the beginning of any innovation project through to sustaining and disseminating project findings.[16,19–21]

While participants had higher *inform* scores than *involve* and *empower* scores, both the CFA and regression analysis identified that participants felt more engaged when they were involved. Used alone, informing stakeholders is an insufficient strategy if engagement is the desired outcome. One-way dissemination of information has been regarded as passive participation in other models of participation, as there are no assurances that stakeholders’ views on an issue will be considered without the venue for those views to be expressed.[42] One could theorize that being informed is a prerequisite to being involved or participating in final decision making; however, further research needs to be conducted to determine if *inform* is an empirical precursor to *involve*.

Based on both the *not applicable* responses and group comparisons, healthcare professionals viewed engagement differently depending on the governance level within which they work. Not surprisingly, SCN leaders at the top of the hierarchy and accountability structures, and often the initiators of engagement, were the most engaged group. This was in direct comparison to the frontline stakeholders who felt much less engaged, with mean scores ranging from 1.74 to 2.69. Engaging frontline healthcare professionals in quality improvement has been an issue that remains concerning for health systems globally,[2,23,66–68] Interestingly, SCN members and support staff who work more peripherally with the SCNs did not differ in their engagement scores, although support staff more often reported that items were not applicable, particularly *empower* items.

This tool can provide a mechanism to quickly assess the dimensions of engagement in clinical networks, and to help networks evaluate the intended results of engagement efforts. Before use, however, organizations are encouraged to pilot test the questionnaire, as context between locations may differ thus yielding different results due to missing questions (referred to as construct underrepresentation). This would allow for context specific questions to be developed in order to best capture engagement. Results of this study suggest that we need to further explore engagement in targeted stakeholder groups and over time. Moreover, further study is required to determine the organizational and individual barriers facing particular groups. This could lead to mapping more effective strategies to ameliorate low engagement in targeted groups (e.g., opinion leaders, champions, targeted messaging, organizational interventions, education, financial incentives). Based on the moderate-to-low engagement scores of frontline stakeholders, the direction and strategies to enhance practitioner engagement should be reconsidered within this health system context. Hess and colleagues[69] suggest that engagement strategies that are embedded in the cultural context of the organization, that enhance interactivity among team members, and that build social learning spaces and processes (audit and feedback) offer opportunities for teams to meet their goals and have ownership of improvements. For SCN leaders, this will require examining existing communication and knowledge pathways between the SCN members (clinicians, patient representatives, leaders, operational leaders), support staff, and frontline end-users to formally and informally exchange ideas for improvement of implementation of existing SCN initiatives, as well as inform development of other initiatives where practice gaps exist.

### Limitations

This study has several limitations. First, we acknowledge the potential for response bias from the self-report survey approach; however, we believe that social desirability was unlikely given the range of item scores and means exhibited. Second, we used a self-selected convenience sample and were not able to compare responders to non-responders, which may have led to significant selection bias. Our intention was to sample diverse healthcare stakeholders—from frontline providers to the executive teams. We know from a comprehensive scoping review (in progress) that these networks globally connect many relevant stakeholder groups; indeed, this is the a key aim of these networks globally. However, our study included stakeholdersconnected to nine SCNs undertaking specific project in Alberta, Canada. This limits the generalizability of our findings to other SCNs, healthcare organizations,, activities, and low- and middle-income countries. We also recognize that further work needs to be done to confirm our findings outside of clinical networks, and with larger samples, to replicate the pattern of engagement across governance levels and professional groups. Moreover, measurement of engagement has to take into consideration the degree of maturation of the network. In the SCNs studied, there were variations in their length of operation and degree of maturation. Lastly, the *not applicable* response option may have been interpreted in various ways by the participants, including expectations around engagement (“I did not expect to be engaged, so rated *not applicable*”) or very recent/new involvement with the networks (“I do not know enough about what has been going on, so rated *not applicable*).

## Conclusion

To conclude, this clinical networks engagement tool demonstrates preliminary evidence of construct validity and reliability. In further work, we propose to assess engagement in broader network activities beyond that of discrete projects, as well as evaluating the factors that influence engagement and how the maturity of networks factors into engagement.

## Supporting information

S1 File Original Item Descriptive Statistics and Exploratory Factor Analysis, and Item Not Applicable Responses by Governance Level. (PDF)

S2 File Final Clinical Networks Engagement Tool. (PDF)
