# Supplementary material for: Development and psychometric testing of the clinical networks engagement tool
Source: PLoS One. 2017 Mar 28;12(3):e0174056. doi: 10.1371/journal.pone.0174056 (PMC5369681; doi:10.1371/journal.pone.0174056)
Supplement: S1 File — (PDF) [file pone.0174056.s001.pdf]

S1 File. Original Item Descriptive Statistics and Exploratory Factor Analysis, and Item Not Applicable Responses by Governance Level

Table A Item Descriptive Statistics

Items			M	SD	Missing %	N/A %
**Global engagement**						
Q1.	glob_prio	How engaged have you been in the following activities: Setting SCN priorities	2.62	1.32	0.9	-
Q2.	glob_plan	How engaged have you been in the following activities: Planning SCN projects	2.70	1.33	0.2	-
Q3.	glob_impl	How engaged have you been in the following activities: Implementing SCN projects	2.77	1.42	1.2	-
Q4.	glob_eval	How engaged have you been in the following activities: Evaluating SCN projects	2.46	1.37	1.4	-
**Inform**						
Q5.	inf_prio	I have been provided with information about SCN priorities	3.91	1.18	0.9	3.3
Q6.	inf_plan	I have been provided with information about how SCN projects are planned	3.50	1.27	0.5	3.5
Q7.	inf_impl	I have been provided with information about how SCN projects are implemented	3.51	1.26	0.9	3.8
Q8.	inf_eval	I have been provided with information about how SCN projects are evaluated	3.35	1.25	1.4	3.1
Q9.	inf_a	I received information to help me understand the overall decision-making process of the SCN projects	3.44	1.23	0.9	5.4
Q10.	inf_b	I received information to help me understand the problems, alternatives, and/or solutions of the SCN projects	3.33	1.23	0.7	5.2
Q11.	inf_c	The information I received was high quality, consistent, timely, appropriate, and easily understood	3.38	1.16	0.7	7.5
**Consult**						
Q12.	cons_prio	I have had an opportunity to provide feedback about SCN priorities	3.47	1.31	1.2	8.5
Q13.	cons_plan	I have had an opportunity to provide feedback about how SCN projects are planned	3.22	1.23	1.7	6.8
Q14.	cons_impl	I have had an opportunity to provide feedback about how SCN projects are implemented	3.25	1.25	2.1	8.0
Q15.	cons_eval	I have had an opportunity to provide feedback about how SCN projects are evaluated	3.14	1.23	2.1	7.8
Q16.	cons_a	I have had an opportunity to provide feedback during the SCN decision-making process	3.58	1.28	2.1	6.4
Q17.	cons_b	My feedback about SCN projects has been listened to and acknowledged	3.63	1.18	1.7	11.1
Q18.	cons_c	I have been provided with information about how my input influenced decisions about SCN projects	3.11	1.21	2.1	10.4
Q19.	cons_d	If my input about SCN projects was not considered, I understand why	3.20	1.13	2.4	18.4
**Involve**						
Q20.	invo_prio	The SCN has worked with me to ensure my concerns and issues have been consistently understood and considered for setting SCN priorities	3.30	1.21	2.1	13.4
Q21.	invo_plan	The SCN has worked with me to ensure my concerns and issues have been consistently understood and considered for planning SCN projects	3.32	1.20	2.6	12.0

Q22.	invo_impl	The SCN has worked with me to ensure my concerns and issues have been consistently understood and considered for implementing SCN projects	3.31	1.18	2.6	12.3
Q23.	invo_eval	The SCN has worked with me to ensure my concerns and issues have been consistently understood and considered for evaluating SCN projects	3.24	1.18	3.3	13.7
Q24.	invo_a	My concerns and issues have been directly reflected in the alternatives developed for SCN projects	3.23	1.10	2.4	17.9
Q25.	invo_b	I have been provided with information about how my concerns and issues influenced decisions about SCN projects	3.08	1.12	3.1	16.3
Q26.	invo_c	My concerns and issues have been adequately considered in SCN decision making	3.23	1.12	3.5	16.7
**Collaborate**						
Q27.	coll_prio	The SCN partnered with me throughout the processes of setting SCN priorities	3.28	1.27	3.1	11.6
Q28.	coll_plan	The SCN partnered with me throughout the processes of planning SCN projects	3.35	1.22	2.6	10.4
Q29.	coll_impl	The SCN partnered with me throughout the processes of implementing SCN projects	3.33	1.21	2.8	12.0
Q30.	coll_eval	The SCN partnered with me throughout the processes of evaluating SCN projects	3.20	1.19	3.5	13.0
Q31.	coll_a	I have been involved in each aspect of SCN decision making	2.80	1.20	3.5	9.0
Q32.	coll_b	I have had an opportunity to develop alternatives for SCN projects	3.04	1.21	3.5	11.3
Q33.	coll_c	I have had an opportunity to identify a preferred solution for SCN projects	3.18	1.22	4.0	10.4
Q34.	coll_d	My alternatives and recommendations have been incorporated into SCN decisions to a large extent	3.03	1.14	4.2	12.3
**Empower**						
Q35.	empo_prio	I have been given final decision-making authority about SCN priorities	2.19	1.10	3.5	22.4
Q35.	empo_plan	I have been given final decision-making authority about how SCN projects are planned	2.23	1.11	3.5	22.2
Q37.	empo_impl	I have been given final decision-making authority about how SCN projects are implemented	2.27	1.12	3.8	21.5
Q38.	empo_eval	I have been given final decision-making authority about how SCN projects are evaluated	2.25	1.08	4.0	21.2
Q39.	empo_a	Decisions I made about SCN projects have been implemented to a large extent	3.04	1.11	6.6	20.3
Q40.	empo_b	I am accountable for the decisions made about SCN projects	3.01	1.31	3.8	19.6

Note: N/A – not applicable.

Table B. Original Exploratory Factor Analysis

Items and Subscales	Rotated Factor Loadings					
	1	2	3	4	5	6
1. Involve						
invo_prio	**0.54**	0.21	0.02	0.02	0.27	0.11
invo_plan	**0.64**	0.17	0.02	0.02	0.23	0.13
invo_impl	**0.63**	0.08	0.06	0.08	0.27	0.14
invo_eval	**0.57**	0.09	0.10	0.08	0.29	0.13
coll_prio	**0.72**	0.06	0.02	0.01	0.09	-0.10
coll_plan	**0.79**	-0.05	0.03	0.07	0.06	-0.17
coll_impl	**0.81**	-0.14	0.07	0.11	0.08	-0.12
coll_eval	**0.77**	-0.12	0.11	0.06	0.06	-0.17
coll_a	**0.58**	0.23	0.20	0.05	-0.08	-0.03
coll_b	**0.70**	0.19	0.13	-0.01	-0.14	-0.06
coll_c	**0.65**	0.17	0.14	0.03	-0.04	-0.03
coll_d	**0.46**	0.16	0.26	0.03	0.06	0.04
2. Inform						
inf_prio	0.00	**0.81**	0.01	0.00	0.05	-0.01
inf_plan	0.05	**0.85**	-0.01	0.10	-0.05	0.02
inf_impl	-0.08	**0.79**	0.11	0.20	-0.02	0.01
inf_eval	-0.10	**0.69**	0.09	0.24	0.06	-0.05
inf_a	0.07	**0.79**	0.02	-0.05	0.04	-0.09
inf_b	0.14	**0.73**	0.07	-0.05	0.02	-0.08
inf_c	0.03	**0.50**	0.02	-0.09	0.23	-0.12
cons_a	0.15	**0.49**	0.01	0.05	0.11	-0.23
3. Empower						
empo_prio	0.00	0.02	**0.96**	-0.02	-0.02	-0.02
empo_plan	0.01	0.00	**0.98**	0.01	-0.03	-0.03
empo_impl	0.00	0.00	**0.99**	0.02	-0.03	0.00
empo_eval	0.03	0.01	**0.97**	0.01	-0.05	0.00
empo_a	0.16	0.02	**0.44**	-0.08	0.31	0.07
empo_b	0.08	0.05	**0.46**	0.01	0.29	0.03
4. Global						
glob_prio	0.13	0.30	-0.13	**0.48**	-0.03	0.03
glob_plan	0.15	0.12	-0.09	**0.73**	-0.05	0.03
glob_impl	-0.02	-0.05	0.07	**0.83**	0.01	-0.01
glob_eval	-0.08	-0.06	0.04	**0.90**	0.07	-0.06
5. Considered						
cons_b	-0.08	0.16	0.04	0.02	**0.68**	-0.19
cons_c	-0.02	0.04	0.08	0.06	**0.68**	-0.27
cons_d	-0.04	0.04	0.04	0.06	**0.72**	-0.09
invo_a	0.28	0.02	0.08	0.04	**0.63**	0.14
invo_b	0.26	0.01	0.06	-0.01	**0.68**	0.08
invo_c	0.28	0.04	0.00	0.02	**0.71**	0.17
6. Consult						
cons_prio	0.27	**0.40**	-0.09	-0.06	0.10	**-0.40**
cons_plan	0.26	0.29	-0.03	0.09	0.09	**-0.52**
cons_impl	0.17	0.19	0.09	0.13	0.14	**-0.54**
cons_eval	0.20	0.16	0.10	0.11	0.12	**-0.58**
Eigenvalue	20.54	4.02	2.12	1.83	1.40	1.05
% of variance	51.34	10.05	5.31	4.56	3.50	2.63
Cronbach's α	0.97	0.94	0.95	0.87	0.93	0.94

Note: 77.4% of the variance explained; rotation converged in 17 iterations; bold font indicates item factor loadings.

Table C Item *Not Applicable* Responses by Governance Level

Item	Leaders		Members		Support		Stakeholders		X^2 (3)	*p*
	n	%	*n*	%	*n*	%	*n*	%		
inf_prio	2	2.8	3	1.3	3	6.1	6	9.4	11.61 [a]	.006
inf_plan	2	2.8	3	1.3	3	6.0	7	10.8	14.43 [a]	.002
inf_impl	2	2.8	4	1.7	3	6.0	7	10.8	12.25 [a]	.014
inf_eval	2	2.8	2	0.9	3	6.0	6	9.5	13.96 [a]	.002
invo_prio	6	8.7	24	10.3	10	20.0	17	27.4	15.32	.002
invo_plan	5	7.1	23	10.0	7	14.0	16	25.8	13.47 [a]	.003
invo_impl	3	4.3	24	10.4	9	18.0	16	25.8	16.57 [a]	.007
invo_eval	4	5.7	25	10.9	13	27.1	16	25.8	19.69 [a]	< .001
empo_prio	10	14.5	39	16.9	25	52.1	21	34.4	34.87	< .001
empo_plan	10	14.5	38	16.5	24	50.0	22	35.5	33.50	< .001
empo_impl	9	13.0	37	16.1	23	47.9	22	36.1	33.38	< .001
empo_eval	9	13.0	37	16.2	22	45.8	22	36.1	30.59	< .001

Note: [a] Fisher's exact test
