# Supplementary material for: Development and psychometric testing of the clinical networks engagement tool
Source: PLoS One. 2017 Mar 28;12(3):e0174056. doi: 10.1371/journal.pone.0174056 (PMC5369681; doi:10.1371/journal.pone.0174056)
Supplement: S2 File — (PDF) [file pone.0174056.s002.pdf]

S2 File. Final Clinical Networks Engagement Tool

Welcome!

You are important to the **Strategic Clinical Network(s) (SCN)** and their work.

As part of a larger study about SCN effectiveness, we are conducting a study to understand engagement in SCNs across Alberta. We want to know about your level of engagement in SCN projects, how the SCN has engaged you, and your beliefs about engaging in SCN projects.

IMPORTANT:

- Your participation in this questionnaire is voluntary.
- By submitting the questionnaire, your participation implies consent. Results will be reported in aggregate form so that individual results will not be directly identifiable.
- All data will be stored at the University of Calgary and will only be accessible to this study's researchers.

Questionnaire:

- The questionnaire will take approximately 15-20 minutes to complete. Some of the questions may seem to be repeated.
- There are slight differences in wording that are intended to help us understand what individuals think about engagement.

Thank you so much for your time!

ENGAGEMENT

This portion of the survey asks about your OVERALL experiences engaging in the SCN.

If you are a member of multiple groups (i.e., committees, working groups), please respond **generally** about your experiences across the groups.

How engaged how you been in the following activities:

	Not at all engaged	Slightly engaged	Moderately engaged	Very engaged	Extremely engaged
Setting SCN priorities	○	○	○	○	○
Planning SCN projects	○	○	○	○	○
Implementing SCN projects	○	○	○	○	○
Evaluating SCN projects	○	○	○	○	○

I have been provided with INFORMATION about:

	Strongly Disagree	Disagree	Neutral	Agree	Strongly Agree	N/A
SCN priorities	○	○	○	○	○	○
how SCN projects are planned	○	○	○	○	○	○
how SCN projects are implemented	○	○	○	○	○	○
how SCN projects are evaluated	○	○	○	○	○	○

How much do you agree with the following statements:

	Strongly Disagree	Disagree	Neutral	Agree	Strongly Agree	N/A
I received information to help me understand the overall decision-making process of the SCN projects	○	○	○	○	○	○
I received information to help me understand the problems, alternatives, and/or solutions of the SCN projects	○	○	○	○	○	○
The information I received was high quality, consistent, timely, appropriate, and easily understood	○	○	○	○	○	○

I have had an opportunity to provide FEEDBACK about:

	Strongly Disagree	Disagree	Neutral	Agree	Strongly Agree	N/A
SCN priorities	○	○	○	○	○	○
how SCN projects are planned	○	○	○	○	○	○
how SCN projects are implemented	○	○	○	○	○	○
how SCN projects are evaluated	○	○	○	○	○	○

How much do you agree with the following statements:

	Strongly Disagree	Disagree	Neutral	Agree	Strongly Agree	N/A
I have had an opportunity to provide feedback during the SCN decision-making process	○	○	○	○	○	○
My feedback about SCN projects has been listened to and acknowledged	○	○	○	○	○	○
I have been provided with information about how my input influenced decisions about SCN projects	○	○	○	○	○	○
If my input about SCN projects was not considered, I understand why	○	○	○	○	○	○

The SCN has WORKED WITH ME to ensure my concerns and issues have been consistently understood and considered for:

	Strongly Disagree	Disagree	Neutral	Agree	Strongly Agree	N/A
setting SCN priorities	○	○	○	○	○	○
planning SCN projects	○	○	○	○	○	○
implementing SCN projects	○	○	○	○	○	○
evaluating SCN projects	○	○	○	○	○	○

How much do you agree with the following statements:

	Strongly Disagree	Disagree	Neutral	Agree	Strongly Agree	N/A
My concerns and issues have been directly reflected in the alternatives developed for SCN projects	○	○	○	○	○	○
I have been provided with information about how my concerns and issues influenced decisions about SCN projects	○	○	○	○	○	○
My concerns and issues have been adequately considered in SCN decision making	○	○	○	○	○	○

The SCN PARTNERED with me throughout the processes of:

	Strongly Disagree	Disagree	Neutral	Agree	Strongly Agree	N/A
setting SCN priorities	○	○	○	○	○	○
planning SCN projects	○	○	○	○	○	○
implementing SCN projects	○	○	○	○	○	○
evaluating SCN projects	○	○	○	○	○	○

How much do you agree with the following statements:

	Strongly Disagree	Disagree	Neutral	Agree	Strongly Agree	N/A
I have been involved in each aspect of SCN decision making	○	○	○	○	○	○
I have had an opportunity to develop alternatives for SCN projects	○	○	○	○	○	○
I have had an opportunity to identify a preferred solution for SCN projects	○	○	○	○	○	○
My alternatives and recommendations have been incorporated into SCN decisions to a large extent	○	○	○	○	○	○

	Strongly Disagree	Disagree	Neutral	Agree	Strongly Agree	N/A
SCN priorities	○	○	○	○	○	○
how SCN projects are planned	○	○	○	○	○	○
how SCN projects are implemented	○	○	○	○	○	○
how SCN projects are evaluated	○	○	○	○	○	○

How much do you agree with the following statements:

	Strongly Disagree	Disagree	Neutral	Agree	Strongly Agree	N/A
Decisions I made about SCN projects has been implemented to a large extent	○	○	○	○	○	○
I am accountable for the decisions made about SCN projects	○	○	○	○	○	○
